# Intimate Partner Violence Against Women in Burkina Faso During COVID-19: A Cross-Sectional Study

**DOI:** 10.1177/00469580251345386

**Published:** 2025-06-24

**Authors:** Simona Skandro, Till Bärnighausen, Michael Lowery Wilson

**Affiliations:** 1 University of Heidelberg, Heidelberg, Germany

**Keywords:** intimate partner violence, COVID-19, pandemic, psychological violence, sexual violence, physical violence, alcohol

## Abstract

The aim of this cross-sectional study is to explore the prevalence of Intimate Partner Violence (IPV) and associated sociodemographic factors in Burkina Faso during the COVID-19 pandemic. We used the 2021 Burkina Faso Demographic and Health Survey (DHS). The total sample counted 9702 eligible women of reproductive age (15-49 years). A 2-stage probability sampling design was used. We compared the prevalence of IPV in 2021 with already published prevalence rates in Burkina Faso from 2010. We applied simple bivariate and multiple logistic regression analyzes to find significant predictors of IPV. A *P*-value < .05 was considered significant. The respondents had a mean age of 31 years, with 2/3 from a rural area and 2/3 from uneducated/illiterate background. The overall prevalence of IPV in Burkina Faso was higher in 2021 than in 2010 (29% vs 15%). The most prevalent form was emotional violence (25.6%), followed by physical (14%) and sexual abuse (3.7%). A respondent’s use of violence toward her partner, having seen her father beat her mother, her partner’s use of alcohol, disagreement about the number of children wanted, and being the first among co-wives were significantly associated with at least one form of IPV. Not working, having an educated partner, rural residence and a smaller household size were negatively associated with at least one type of IPV. This study reveals a high prevalence of IPV in Burkina Faso during the COVID-19-pandemic in 2021 and uncovers associated factors at that time. We recommend future research after the effects of the pandemic have subsided to evaluate whether the increased prevalence was a general or a COVID-19-related trend. Campaigns which aim to reduce IPV should focus on couples with socio-demographic characteristics that are associated with IPV.

**Highlights**

- Compared to 2010, the overall prevalence of IPV in Burkina Faso during COVID-19 in 2021 was almost twice as high (15% vs 29%).
- Women who were not working during the COVID-19 pandemic in 2021 reported less violence by their intimate partner.
- Education of men seem to be protective in the context of Intimate Partner Violence, whereas the educational level of women does not have a significant effect.
- The first among co-wives within polygamous unions reported more intimate partnership violence compared to the remaining spouses.

## Introduction

Intimate Partner Violence (IPV) is a worldwide public health concern which can have devastating and permanent effects on those who experience it. According to the United Nations IPV is defined as a “pattern of behavior in any relationship that is used to gain or maintain power and control over an intimate partner.”^1^

Violence between intimate partners can be expressed in a variety of ways including physical, sexual, emotional and economic abuse. Physical violence can be defined as a deliberate “aggressive or violent behavior by one person toward another that results in bodily injury.”^2^ Sexual abuse is formally recognized as “any nonconsensual or exploitive sexual behavior or activity imposed on an individual without their consent.”^2^ Emotional abuse can be identified as “nonphysical acts that are detrimental to behavioral and affective functioning and overall mental well-being including verbal abuse; intimidation and terrorization; humiliation and degradation; exploitation; harassment; rejection and withholding of affection; isolation; and excessive control”.^2^ Economic abuse can mean the restriction of financial resources or denying access to education or the labor market.^3^

In general, IPV can be directed against men as well as women. While the number of men who experience partner violence is comparable to that of women,^4^ violent acts against women are generally more severe, as they more likely suffer from injuries.^5,6^ In our study we focus on violence against women.

Finding out the true prevalence of IPV is challenging, because it depends on regional, cultural and methodological factors and because victims tend to under-report. It is estimated that globally around 27% of women have suffered from sexual and/or physical violence by their intimate partner at least once in their lifetimes.^7^ According to an international meta-analysis psychological violence is the most prevalent form of IPV, followed by physical and sexual violence.^8^

The estimated prevalence of IPV differs from region to region and is higher in countries which are classified as being low- and middle-income. According to the most recent WHO report the highest prevalence of IPV can be found in Oceania (39%-51%), followed by Southern Asia (35%) and regions in Africa (30%-33%). Regions with the lowest rates include Europe (16%-23%), the rest of Asia (18%-21%), Australia and New Zealand (23%) and North and South America (25%).^7^

Prior research suggests that the consumption of alcohol or other drugs, a history of having experienced or witnessed abuse during childhood, a young age, and a low socioeconomic status are associated with IPV.^9-13^ In world regions where being married to multiple wives is legal and common, IPV is found more often in polygamous relationships than in those which are monogamous.^12,14-16^ Furthermore, working, having attitudes toward wife-beating and a partner with controlling behavior is associated with IPV.^13^

Education, being married and wealth seem controversial in the context of IPV. While some literature counts low education^11,13-15,17^ and being unmarried among the risk factors^9^ others found out that the likelihood of experiencing IPV increases with being married^18^ or more educated.^18,19^ In the same way, the influence of wealth and the type of residence (urban vs rural) on the occurrence of IPV varies considerably.^20,21^Among known protective factors are being older,^9,12,15^ having supportive social networks,^22^ participating in household decisions and being Muslim.^13^

IPV is associated with a number of negative effects on women’s and their children’s physical, mental and social state. Women who experience IPV are more likely to suffer from injuries, chronic pain, infections from sexually transmitted diseases, suicidal behavior, depression, post-traumatic stress disorder, anxiety, and negative impacts from drug abuse.^17^

Negative effects on children include miscarriage among the unborn and a lower birth weight when their mothers experience violence during pregnancy.^17^ Also common are negative impacts on the motor and cognitive development of children and an increased likelihood of suffering child abuse from their parents.^23^

The COVID-19 pandemic and resulting lockdowns may have exacerbated the occurrence of violence within domestic spaces. Previous studies have revealed an increased prevalence of IPV,^24^ whereas others reported no change or a decrease.^24-26^ In some cases an increased severity of IPV during the pandemic was reported.^24,26^

In Burkina Faso, the first case of Coronavirus-19 was detected on March 9, 2020.^27^ In the subsequent period a relevant part of the population had to face intense burdens, such as the delay of needed healthcare visits and limited supply of food and financial resources.^28^

Although data on IPV against women already exist from different countries^13,14^ and earlier times from Burkina Faso.^29^ there is a research gap on recent developments in Burkina Faso. We believe that personal and interpersonal challenges during the COVID-19-pandemic could have had a significant effect on the prevalence of IPV. Our objective is to assess factors that appear controversial in previous studies (see above) and factors which were specifically associated with IPV during the time of the COVID-19-pandemic.

## Methods

### Study Area

Burkina Faso is a country located in the western part of sub-saharan Africa with an estimated population of 22 million in the year 2021.^30^ The majority of the population is Muslim.^31^ The gender ratio is 99 men to 100 women (0.99), which is lower than the global average.^32^ In 2019, Burkina Faso ranked in the lowest 10% on the Gender Inequality Index of the United Nations Development Program.^33^ Legally, the country prohibits domestic violence. However, access to social protection and legal remedies is challenging. Traditional roles are prominent and IPV often a stigmatized issue.^16^

### Data Source and Sampling

The data was collected during the latest DHS Program in Burkina Faso (DHS-VIII), which took place from July 2021 to December 2021.^34^ The sample is based on a 2-stage cluster sampling. In the first stage, so-called enumeration areas (EA) were chosen, which are based on a recent population census and contain a number of households. The second stage served to select a fixed number of households. The questions which provided the information of interest stem from the Woman’s Questionnaire (WQ), the Household’s Questionnaire and the “Domestic Violence Module” (DVM). All data are de-identified by removing personal information that can directly identify individuals. The data is publicly available upon request on the DHS website.^35^ Data access is only granted to researchers who agree to treat it confidentially, to make no effort to identify any individual, household, or enumeration area in the survey and who agree not to publish results in which communities or individuals can be identified.^36^ DHS surveys undergo a validation process, which is regularly updated according to international recommendations and results from validation studies. The validation process includes Pilot tests, Pre-tests, the use of procedures and samples which reproduce conditions during fieldwork, cognitive interviewing and feedback from interviewers and other survey members.^37,38^

### Sample Size, Inclusion and Exclusion Criteria

**Figure 1.**
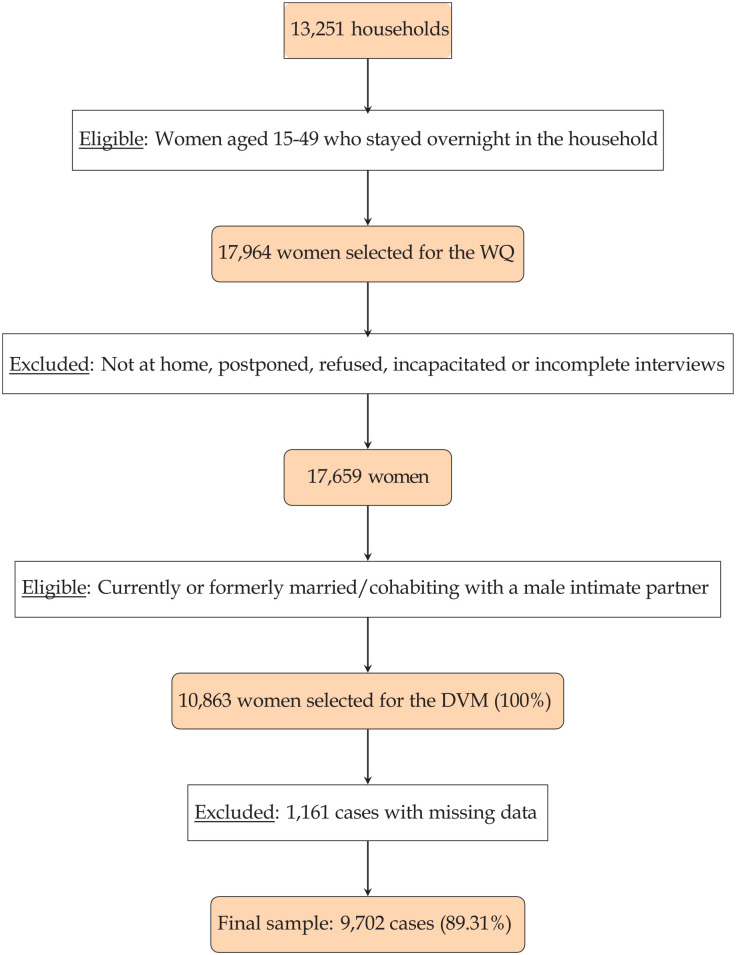
Flowchart of the sample selection process.

Calculation of the sample size indicates that with the estimated population size in Burkina Faso^30^ 384 surveys are needed to have a confidence level of 95% that the real prevalence of IPV is within ±5% of the surveyed value (*P* = .5). In total 13 251 households completed the questionnaires. Excluded were cases in which no competent respondents were available, households with permanently postponed or refused interviews and households for which the dwelling was not found. From the selected households women from 15 to 49 years who stayed in the household the night before the survey were selected for the woman’s questionnaire. Excluded were women who were not at home, who continuously postponed, refused or never completed the interview and whose interview could not be completed due to incapacitation or other reasons. The survey response rate was 98.3%.^39^ In overall the sample size of female respondents counted 17 659. Eligibility for the DVM was given for all female respondents between 15 and 49 years of age who were currently or formerly married or cohabitant with a male intimate partner. Out of the eligible women, 1 per household was randomly selected. In total, 10 863 women were chosen for the DVM. Out of those, 1161 (10.69%) respondents were excluded due to missing data. The final sample size counted 9702 cases (89.31%), which is above the calculated sample size. Figure 1 shows how the number of study participants was reached.

### Measures

Every variable of interest was either retrieved or constructed from the publicly available data in the DHS files. The outcome measures are defined as self-reported physical, sexual, or psychological abuse perpetrated by a current or former intimate partner. The questions from the DVM which served to asses the 3 types of IPV are presented in Table 1.

**Table 1.** Questions in the Domestic Violence Module of the 2021 Burkina Faso DHS.

Type of violence	Question
Emotional violence	Does/did your (last) husband/partner ever (1) say or do something to humiliate you in front of others? (2) threaten you or someone close to you with harm? (3) insulted you or made you feel bad?
Sexual violence	Does/did your (last) husband/partner ever (1) physically force you to have sexual intercourse with him even when you did not want to? (2) force you into other sexual acts you did not want to?
Physical violence	Does/did your (last) husband/partner ever (1) push you, shake you, or throw something on you? (2) slap you? (3) punch you with his fist or with something that could hurt you? (4) kick you or drag you? (5) try to strangle you or burn you? (6) threaten you with a knife, gun or other type of weapon? (7) twisted your arm or pulled your hair? Did the following ever happen because of something your (last) husband/partner did to you: (8) You had bruises and aches? (9) You had eye injuries, sprains, dislocations or burns? (10) You had wounds, broken bones/teeth or other serious injury?

Emotional abuse was identified when the respondent answered “yes” to 1, 2, or 3 of the afore-mentioned questions. Depending on the number of affirmative responses, a score ranging from 1 to 3 was assigned and subsequently recoded as “1.” If all 3 questions were answered with “no,” it was recoded as “0.” Sexual violence was positive if the respondent answered “yes” to at least 1 of the 2 questions, resulting in a score of 1 to 2. This was then recoded as “1”; otherwise as “0.” In case of physical abuse, a sum ranging from 1 to 10 was recoded as “1,” while a score of 0 indicated that the woman denied physical abuse from her partner. To determine whether a respondent had experienced “any kind of IPV,” the total score was considered. If she had encountered at least 1 sub-type of IPV, the score was recoded as “1.” If the respondent answered negatively to all questions outlined in Table 1, the total score was “0.”

The factors which were chosen as independent variables are socio-demographic characteristics which have been previously linked to domestic violence in a similar cultural context.^18,40,41^ The factors which were chosen as independent variables can be categorized into 4 subgroups.

#### Women’s Characteristics

Age (in years), occupation (not working/white collar/blue collar), literacy (illeterate/literate), education (none/primary/secondary/higher), tobacco use (yes/no), number of children born, rank among co-wives (first/second or more), use of violence toward partner (yes/no), witnessing her father beat her mother (yes/no).

#### Men’s Characteristics

Age (in years), occupation (not working/white collar/blue collar), alcohol use (yes/no), education (none/primary/secondary/higher).

#### Couples’ Characteristics

Age difference (respondent is older/younger/same age), salary difference (respondent earns more/less/the same), educational difference (higher/lower/same educational level), relationship status (currently/formerly in union), polygyny (yes/no), cohabitation duration (0-9/10-19/>20 years), desire for children (same/different amount of children wanted), partner being present during the interview (yes/no).

#### Households’ Characteristics

Place of residence (rural/urban), wealth quintile (poorest/poorer/middle/richer/richest), household size, number of children in the household under 5 years, sex of household head (male/female), age of household head (in years).

A white collar job is defined as “work that needs mental rather than physical effort”^42^ and includes professionals, clerks, sales, services, and skilled jobs. Blue collar jobs are based on “strength or physical skill rather than office work”^42^ like agricultural, domestic and unskilled jobs. Literacy in the DHS Program is defined as the ability to read a sentence fully or partly.^37^ The wealth index in DHS is defined by the household’s flooring, utilities, ownership of durable goods, land, and livestock (in rural settings).^43^

### Data Analysis

Taking over- and under-sampling of specific population groups into account, the survey design method and sampling weights provided by DHS were used. An unweighted design approach was adopted only for continuous variables and numerical proportions. Initially, a subset of the data was generated comprising respondents who completed the DVM, totaling 9702 participants. In addition, cases which contained unspecific replies like “don’t know” or “no husband information” were excluded. Women’s and men’s characteristics were quantified in numbers (n) and weighted proportions (%). Continuous factors such as age, number of children and household members were presented as weighted means. The couple’s age difference resulted by subtracting the woman’s age from her partner’s age, then by classifying the result into groups based on whether the respondent was older, younger or in the same age group (±2 years) as her partner. In a similar approach we evaluated the educational difference between partners.

The outcome measures were the lifetime experiences of emotional, sexual and/or physical IPV. These experiences were presented as prevalence rates, representing the weighted proportions of female respondents reporting any form of IPV. Survey versions of t-test and chi-square bivariate test were used to determine how strong the independent and dependent variables were associated. A *P*-value < .05 was deemed significant. The variables which were significantly associated with at least 1 type of IPV were chosen for the final regression models. We conducted 4 multi-variable logistic regression models: 1 for every sub-form of IPV and 1 for any form of IPV. To ensure robustness and reliability of the regression models, an examination of multicollinearity was performed using the generalized Variance Inflation Factor (VIF). The variable “polygyny” was redundant in models which included information on the rank among co-wives. The variable “relationship status” was collinear with other socio-demographic factors. Therefore these variables were removed. The models’ coefficients were exponentiated and shown as odds ratios (ORs) along with the 95% Confidence Intervals (CI). Model fitness was assessed using McFadden’s pseudo *R*². The model on sexual IPV showed a moderate fit (pseudo *R²* = .34), while the results for the remaining 3 models indicated a strong fit (pseudo *R*² values between .86 and .87). The analyses were conducted with RStudio version 2023.12.1 + 402 for macOS, utilizing the “survey” package for the survey design method.

### Ethical Consideration

The data incorporated in this research are accessible on the DHS website.^35^ Surveys utilized for DHS assessments underwent scrutiny and endorsement by the ICF Institutional Review Board (IRB). Verbal and written informed consent was secured from all participants capable of consenting to or refusing participation in the survey. The ethical approval processes are described on the DHS website.^44^

## Results

### Socio-Demographic Characteristics of the Study Population

Table 2 shows the socio-demographic characteristics of the sample. The 9702 female respondents who fulfilled the inclusion criteria had an average age of 31 years, whereas their partners’ mean age was 42 years. In over 87%, the partner was older than the woman. Around two-thirds lived in a rural area. The wealth quintiles were almost equally represented with 18.9% of women from the poorest and 21.4% from the richest quintile. Similarly, around one-third of the respondents was either not working or performed a white or blue collar job. In 87.0% of the cases, the respondent earned less than her partner. Two-thirds of the women considered themselves illiterate and had no education, whereas almost 3 out of 4 partners (73.4%) were uneducated. Only 3% of the respondents used tobacco at the time of the survey, whereas 18.1% of their partners used alcohol. An average household consisted of 8 household members with an average of 1.6 children under 5 years of age. The household head was mostly male (in 88.1%) and had an average age of 46 years. 86% of the respondents were in union at the time of the survey. 37.9% declared that they lived in a polygamous union, of which 39.3% were the first among co-wives. Around 44.5% lived with their partner for less than 10 years. 0.6% of the women revealed that they used violence against their partner when he was not hurting her and 6.2% reported that they saw their father beating their mother. 45.5% of the couples wanted the same number of children, whereas 54.5% disagreed. In 99.6% of the cases the partner was not present during the “wife beating justified” questions.

**Table 2.** Socio-Demographic Characteristics of the Sample (n = 9702).

Variable	Subgroup	n (crude)	Mean (SD) or % (weighted)
Respondent’s age			31.0 (±0.1)
Partner’s age			41.7 (±0.2)
	Respondent is older	109	1.4%
Age difference	Same age (±2 years)	991	11.3%
	Partner is older	7558	87.4%
Residence	Rural	6348	69.0%
	Urban	3354	31.0%
	Poorest (Q1)	2091	18.9%
	Poorer (Q2)	1872	19.3%
Wealth quintile^a^	Middle (Q3)	1960	20.2%
	Richer (Q4)	1931	20.3%
	Richest (Q5)	1848	21.4%
	Not working	3178	33.8%
Respondent’s occupation	White collar^b^	2954	30.2%
	Blue collar^c^	3510	36.0%
	Not working	813	11.1%
Partner’s occupation	White collar^b^	3137	35.1%
	Blue collar^c^	4551	53.8%
	Respondent earns more	188	8.9%
Salary difference	Both earn the same	114	4.1%
	Partner earns more	2010	87.0%
Respondent’s literacy^d^	Illiterate	6557	67.9%
	Literate	3120	32.1%
	None	6282	65.8%
Respondent’s education	Primary	1303	12.8%
	Secondary	1939	19.4%
	Higher	178	2.1%
	None	5822	73.4%
Partner’s education	Primary	1127	12.3%
	Secondary	1109	11.0%
	Higher	302	3.4%
	She is more educated	1218	13.9%
Educational difference	Same educational level	5823	72.0%
	He is more educated	1319	14.1%
Respondent’s use of tobacco	No	9456	97.0%
	Yes	246	3.0%
Partner’s use of alcohol	No	7781	81.9%
	Yes	1921	18.1%
No. of household members			8.1 (±0.1)
No. of born children			3.3 (±0.0)
No. of children <5 years at home			1.6 (±0.0)
Age of household head			45.7 (±0.2)
Sex of household head	Female	1202	11.9%
	Male	8500	88.1%
Relationship status	Currently in union	8658	86.4%
	Formerly in union	1044	13.6%
Polygyny (having co-wives)	No	6316	62.1%
	Yes	2323	37.9%
Rank among co-wives	First	906	39.3%
	Second or more	1416	60.7%
	0-9 years	4389	44.5%
Cohabitation duration	10-19 years	3136	30.5%
	20+ years	2177	25.0%
Respondent’s use of violence^e^	No	9626	99.4%
	Yes	76	0.6%
Father’s use of violence^f^	No	8716	93.8%
	Yes	653	6.2%
Desire for children	Want the same amount	2552	45.5%
	Want different amounts	2755	54.5%
Partner present for “wife beating justified” questions	No	9660	99.6%
	Yes	42	0.4%

a Based on a household’s flooring material, water source, toilet facilities, ownership (television, car, etc.), adjusted for rural and urban lifestyle.

b Professionals, clerks, sales, services, and skilled jobs.

c Agricultural, domestic, unskilled jobs and others.

d Ability to read parts or whole sentences. Blind and visually impaired were excluded.

e Whether the respondent ever physically hurt her partner when he was not hurting her.

f Whether respondent’s father ever beat her mother.

### Prevalence of IPV and Its Subtypes

In total, 2756 respondents reported the experience of at least one form of violence from their intimate partner, constituting 28.5% of the sample (95% CI = 27.4-30.0). When comparing different types of IPV, emotional violence emerged as the most prevalent with 25.6% of all respondents (95% CI = 24.5-27.0). Approximately 1 out of 7 women (14.0%, 95% CI = 13.2-15.0) reported physical abuse by her partner. Moreover, 3.7% of all participating women stated that they had been coerced into unwanted sexual activity (95% CI = 3.2-4.0).

### Factors Associated With IPV in Burkina Faso

The subsequent factors demonstrated a significant association with every type of IPV without being collinear and were therefore added to all 4 multi-variable regression models: Respondent’s literacy, her education and her partner’s education, his alcohol intake, the woman’s and her father’s use of violence. In addition to the above mentioned variables, the following ones were strongly associated with emotional, physical and any type of IPV at the bivariate level and hence selected for 3 of the final models: both partners’ age and occupation, place of residence, household size, and number of born children, the respondent’s rank among co-wives, cohabitation duration and whether the couple wants the same or a different amount of children.

In case of sexual abuse, in addition to the first mentioned variables, the sex of the household head and polygyny were relevant at the bivariate model and added to the final regression model.

A partner being present for the “wife beating justified” question relevantly reduced the report of physical IPV. Therefore the variable was included in the final regression model on Physical IPV.

Table 3 provides an overview of which factor was significantly associated with which type of IPV at the bivariate level (*P* < .05).

**Table 3.** Factors Significantly Associated With IPV at the Bivariate Level.

Factor	Emotional IPV	Physical IPV	Sexual IPV	Any IPV
Respondent’s literacy	✓	✓	✓	✓
Respondent’s education	✓	✓	✓	✓
Partner’s education	✓	✓	✓	✓
Partner’s alcohol intake	✓	✓	✓	✓
Respondent’s use of violence	✓	✓	✓	✓
Father’s use of violence	✓	✓	✓	✓
Polygyny	✓^a^	✓^a^	✓	✓^a^
Rank among co-wives	✓	✓		✓
Respondent’s age	✓	✓		✓
Partner’s age	✓	✓		✓
Respondent’s occupation	✓	✓		✓
Partner’s occupation	✓	✓		✓
Place of residence	✓	✓		✓
Household size	✓	✓		✓
Number of born children	✓	✓		✓
Cohabitation duration	✓	✓		✓
Desire for children (same/different)	✓	✓		✓
Household head’s sex			✓	
Relationship status			✓^a^	
Partner present for “wife beating justified” question		✓		

a Not included in the multivariable regression models due to multicollinearity.

In order to account for potential age-related effects, another separate multivariate regression model was constructed for sexual IPV, incorporating both the woman’s and man’s age as an independent variable. When adjusting for age no different associations with sexual IPV could be seen compared to the previous model. Regarding the other sub-types of IPV, age-related variables are already included in the first multi-variable regression models.

The results of our multivariate regression analyses showed that the use of violence when not being harmed herself, the partner’s alcohol intake and witnessing violence during childhood are significantly related to the experience of all types of IPV.

Respondents that engaged in violence against their partners without being harmed themselves had the highest ORs for exposure to one or multiple types of IPV compared to the rest of the respondents. The odds ratios here span from 11.014 for emotional abuse (95% CI = 5.443, 22.286) to 25.867 for physical abuse (95% CI = 12.943-51.694). For any type of IPV, respondents that used physical violence against their partners had 18.266 higher odds of being abused than women that denied hurting their partners (95% CI = 7.483-44.588).

Respondents whose partners used to drink alcohol at the time of the survey had 2.136 higher odds of being abused in an emotional way than respondents whose partners did not drink (95% CI = 1.857-2.456). Likewise, they showed 3.087 higher odds of suffering from sexual violence (95% CI = 2.345-4.063), 3.076 higher odds of suffering from physical abuse (95% CI = 2.615-3.619) and 2.302 higher odds for any type of violence (95% CI = 2.007-2.640) in comparison to women whose partners were non-alcoholic. No significant effect of being a female tobacco user on experiencing IPV in Burkina Faso was revealed.

Having witnessed inter-parental violence during childhood is significantly associated with all types of IPV. Compared to respondents without a background of family violence, those whose fathers used physical violence against their mothers showed 2.623 higher odds of being emotionally abused by their intimate partner (95% CI = 2.123-3.240), 3.307 higher odds of being forced into sexual acts (95% CI = 2.273-4.812), 2.645 higher odds of experiencing physical violence (95% CI = 2.068-3.382) and 2.720 higher odds of experiencing any type of violence (95% CI = 2.207-3.352).

The respondent’s occupational status showed a significant correlation with all types of violence except for sexual IPV. In general, women who were working had higher odds of experiencing emotional, physical or any IPV than women who were not. More precisely, those who were doing blue collar jobs had higher odds than those doing white collar jobs. Compared to unemployed women, women doing white collar jobs had 1.474 higher odds for exposure to emotional IPV (95% CI = 1.267-1.717), 1.432 higher odds of experiencing physical IPV (95% CI = 1.183-1.733) and 1.418 higher odds of experiencing any type of IPV (95% CI = 1.226-1.641). Women engaged in blue collar jobs had 1.640 higher odds for the exposure to emotional IPV (95% CI = 1.419-1.894), 1.701 higher odds of experiencing physical abuse (95% CI = 1.418-2.039) and 1.563 higher odds of experiencing any type of abuse (95% CI = 1.360-1.795) compared to women who were not working.

The prevalence ratio of sexual partner violence against women was significantly lower when their partners’ level of education was secondary or higher. The prevalence of sexual IPV among women was almost 40% lower when their partners had a secondary education (OR = 0.606, 95% CI = 0.376-0.979) and more than 90% lower in case of higher education (OR = 0.070, 95% CI 0.017-0.293) compared to no education. Adversely, there was a 1.5% increase in prevalence among women whose partners had primary education compared to no education.

The higher the partner’s educational level was, the lower the odds of experiencing physical IPV. Compared to women with uneducated partners, those whose partners had primary education revealed 14% lower odds of experiencing physical IPV (OR = 0.858, 95% CI = 0.676-1.088), 33% lower odds in case of secondary education (OR = 0.671, 95% CI = 0.520-0.865) and 80% lower odds in case of higher education (OR = 0.200, 95% CI = 0.094-0.426). No significant association between a woman’s educational level or literacy and the experience of IPV in Burkina Faso was found.

The rank among co-wives revealed a significant association with being abused in an emotional or any kind of way. Wives of second or higher rank had almost 40% lower odds of experiencing emotional or any type of abuse compared to the first wives (Odds ratios = 0.615 and 0.645 respectively).

The type of Residence plays a crucial role when it comes to physical abuse. Respondents living in Urban regions had around 20% lower odds of being physically abused compared to those from Rural regions (OR = 0.787, 95% CI = 0.667-0.928).

The total number of household members was relevantly correlated with physical abuse at home. The bigger the household size the higher the odds ratio of being exposed to physical IPV (OR = 1.027, 95% CI = 1.009-1.045).

Disagreement among intimate partners about how many children they want increased the prevalence ratio of emotional violence by around 70% (OR = 1.669, 95% CI = 1.433-1.944), the prevalence ratio of sexual violence by 82% (OR = 1.818, 95% CI = 1.491-2.216) and the prevalence ratio of any type of violence by 68% (OR = 1.676, 95% CI = 1.444-1.946).

The odds of reporting physical violence was almost 80% lower when the respondent’s partner was present during the question on “wife beating” (OR 0.203, 95 CI = 0.056-0.738).

An overview of the predictors of IPV can be found in Table 4. A *P*-value < .05 was considered significant. Nevertheless, predictors associated with IPV at even lower *P*-values were marked as follows: *** (*P* < .001) and ** (*P* < .01).

**Table 4.** Predictors of IPV in Burkina Faso, Presented as Odds Ratios and 95% Confidence Intervals.

Exposure variable	Model 1 Emotional IPV	Model 2 Sexual IPV	Model 3 Physical IPV	Model 4 Any IPV
Respondent’s age	1.016 (1.009, 1.022)		1.023 (1.015, 1.032)	1.013 (1.007, 1.020)
Partner’s age	1.006 (1.001, 1.012)		1.012 (1.005, 1.018)	1.006 (1.001, 1.011)
Residence				
Rural	1		1	1
Urban	0.812 (0.714, 0.924)		0.787* (0.667, 0.928)	0.801 (0.707, 0.907)
Respondent’s occupation				
Not working	1		1	1
White collar	1.474** (1.267, 1.717)		1.432** (1.183, 1.733)	1.418*** (1.226, 1.641)
Blue collar	1.640** (1.419, 1.894)		1.701** (1.418 2.039)	1.563*** (1.360, 1.795)
Partner’s occupation				
Not working	1		1	1
White collar	1.277 (0.996, 1.636)		1.168 (0.872, 1.564)	1.270 (1.002, 1.609)
Blue collar	1.448 (1.139, 1.842)		1.449 (1.097, 1.915)	1.460 (1.161, 1.836)
Respondent’s literacy				
Illiterate	1	1	1	1
Literate	0.770 (0.676, 0.876)	1.378 (1.047, 1.813)	0.758 (0.641, 0.897)	0.803 (0.709, 0.910)
Respondent’s education				
None	1	1	1	1
Primary	1.063 (0.892, 1.267)	1.545 (1.072, 2.225)	1.182 (0.955, 1.463)	1.090 (0.920, 1.292)
Secondary	0.739 (0.632, 0.865)	1.185 (0.856, 1.642)	0.700 (0.564, 0.868)	0.796 (0.685, 0.926)
Higher	0.218 (0.116, 0.409)	0.303 (0.093, 0.985)	0.147 (0.059, 0.366)	0.232 (0.130, 0.414)
Partner’s education				
None	1	1	1	1
Primary	1.099 (0.915, 1.321)	1.015* (0.678, 1.519)	0.858* (0.676, 1.088)	1.087 (0.910, 1.298)
Secondary	0.761 (0.625, 0.926)	0.606* (0.376, 0.979)	0.671* (0.520, 0.865)	0.769 (0.637, 0.929)
Higher	0.391 (0.263, 0.582)	0.070* (0.017, 0.293)	0.200* (0.094, 0.426)	0.347 (0.234, 0.514)
Partner’s use of alcohol				
No	1	1	1	1
Yes	2.136** (1.857, 2.456)	3.087*** (2.345, 4.063)	3.076*** (2.615, 3.619)	2.302** (2.007, 2.640)
No. of. household members	1.018 (1.003, 1.034)		1.027* (1.009, 1.045)	1.022 (1.007, 1.037)
No. of. born children	1.068 (1.044, 1.093)		1.123 (1.091, 1.156)	1.070 (1.045, 1.094)
Sex of household head				
Female		1		
Male		0.615 (0.431, 0.878)		
Polygyny				
No		1		
Yes		1.394 (1.019, 1.906)		
Rank among co-wives				
First	1		1	1
Second or more	0.615** (0.494, 0.767)		0.600 (0.458, 0.788)	0.645* (0.520, 0.801)
Cohabitation duration				
0-9 years	1		1	1
10-19 years	1.568 (1.371, 1.794)		1.512 (1.271, 1.798)	1.537 (1.350, 1.749)
20+ years	1.357 (1.165, 1.580)		1.554 (1.286, 1.878)	1.292 (1.115, 1.497)
Respondent’s use of violence				
No	1	1	1	1
Yes	11.014*** (5.443, 22.286)	22.666*** (12.460, 41.23)	25.867*** (12.943, 51.694)	18.266*** (7.483, 44.588)
Father’s use of violence				
No	1	1	1	1
Yes	2.623*** (2.123, 3.240)	3.307*** (2.273, 4.812)	2.645* (2.068, 3.382)	2.720*** (2.207, 3.352)
Desire for children				
Want the same amount	1		1	1
Want different amounts	1.669*** (1.433, 1.944)		1.818*** (1.491, 2.216)	1.676*** (1.444, 1.946)
Partner present for “wife beating justified” question				
No			1	
Yes			0.203*** (0.056, 0.738)	

Significance codes: “***” < .001; “**” < .01; “*” < .05.

## Discussion

This study’s objective was to find the prevalence and associated factors of IPV in Burkina Faso during the COVID-19-pandemic. In 2021, 29% of female respondents stated that they had been exposed to at least one form of intimate partner violence in their lifetime. Consistent with other sources,^8,13^ emotional violence was the most prevalent form (25.6%), followed by physical (14.0%) and sexual violence (3.7%). In addition, we found that specific women’s, men’s and household characteristics were significantly associated with IPV in Burkina Faso.

The reported prevalence of IPV in Burkina Faso was higher than in countries classified as high- or middle-income^7^ which can be attributable to an interplay of social, cultural and political factors. In particular, a low-income setting may come with economic insecurity and limit women’s capability to leave abusive relationships. Furthermore, norms promoting gender inequality, social and family stigma, and less consistently enforced laws are more prevalent in low-income settings.^8^

This study found a lower prevalence of IPV in Burkina Faso than in other Sub-Saharan African countries such as Togo,^13^ Nigeria,^45^ Kenya,^14^ Angola,^12^ and the African region in general.^7^ It is possible that IPV is less prevalent in Burkina Faso than in her neighboring countries. However, the discrepancies could also be due to under-report in Burkina Faso or due to different research methods used in the above named studies.

Looking at the development of IPV over time, it is striking that in 2010 far less violent acts were reported by women than in 2021.^29^ Compared to a decade before, the documented prevalence of every form of IPV was considerably higher in 2021: physical IPV increased from 11.1% to 14.0%, sexual IPV from 1.5% to 3.7%, emotional IPV from 9.3% to 25.6%. The overall prevalence of IPV almost doubled from 15% to 29%.^21,29^ Given that these estimates are based on data from the DHS program (2010 and 2021 accordingly) in which the same methodology and study design were used, the results can be considered as a reliable assessment of the development over time.

The likelihood of experiencing IPV is significantly shaped by interpersonal family dynamics including childhood experiences and personal habits. Our study indicates that a woman who has witnessed her father physically assault her mother during childhood is more likely to end up with a partner who abuses her in adulthood. This confirms findings from previous studies.^17^ Growing up in a violent home can have devastating effects on children’s development and relevantly shape their behavior.^46^ Possibly, children who repeatedly experience inter-parental violence may develop beliefs which accept or normalize gender-based violence. Likewise, imprinting may play a crucial role where individuals subconsciously develop attractions to (abusive) partners who resemble their (abusive) opposite-sex parent.^47^

We found a notable effect of alcohol on IPV, which is consistent with previous studies from Sub-Saharan Africa.^12,13,21,41^ Alcohol consumption not only correlates with, but actually causes aggressive behavior by “paralyzing” self-control mechanisms and suppressing non-violent responses.^41^

A relevant association between women’s use of violence against their partners and experiencing IPV was found. This evidence is in agreement to an earlier study from Angola.^12^ To clarify that their violent acts were not actually self-defense the respondents were asked whether they ever hurt their partner “when he was not hurting them.” These acts can be seen as a coping mechanism in reaction to stress due to the abuse which they experience. It might also be that women who have a tendency to hurt others more likely find a partner with similar traits.^48^

This study suggests strong evidence that a couple’s background characteristics such as education and occupation have an impact on violence at home. Consistent with previous studies our results indicate that Burkinabe men who are more educated use less violence against their wives.^16^ It is likely that education provides more progressive and egalitarian attitudes toward gender-roles and different ways of non-violent conflict solving. Likewise, one study suggests that an educated population less likely condones violence against women.^49^ Given the significant impact of education, future campaigns may focus on schools and similar institutions to spread awareness of IPV, of its negative effects and of possible support for victims.

Respondents who were not working at the time of the survey reported significantly less emotional, physical or any type of violence. Women who work might challenge the idea of the traditional gender-roles and as a consequence their partners might try to maintain their dominance by using violent ways.^21^ This is consistent with previous research which found that women who feel economically reliant on their partner are less likely to experience physical or psychological violence in Burkina Faso.^16,50^

In contrast to other studies^12,14-16^ this study does not reveal a significant association between polygyny and IPV. However it indicates that when examining polygamous unions alone, the first wife seems to be at a higher risk of being emotionally abused than her co-wives. The time of marriage does not seem to be the contributing factor because no significant impact of the cohabitation duration was found. More likely, the role and expectations given to the first wife differ from those given to her co-wives. If the first wife is chosen by a man’s parents whereas the later wives are more aligned with his personal preferences, conflicts with the first wife might occur. Traditionally the first wife is expected to accept every new co-wife without complaint. In case of resistance she might be confronted with a violent response from her husband. Also, she carries the burden of cumulative stress with every new co-wife who appears. Potentially, a woman who was used to exclusivity with her husband might feel jealousy and competition when new wives are introduced into the family. Furthermore, she has to share the residence’s space and resources with additional persons, which could create tension and willingness for conflict.

The type of residence seems to be a controversial factor in the context of IPV. Our data imply that women who live in urban areas are less likely to experience physical violence by their partner. This is consistent with evidence from some low- and middle-income countries^17^ but inconsistent with findings from other countries in sub-Saharan Africa.^16,21^ This deviation can be due to differences in methodology, design and timing of the studies.

A bigger household size in Burkina Faso seems to contribute to physical violence. An earlier systematic review on IPV published that studies found different effects of family size on IPV.^11^ An increasing number of individuals at home can lead to additional deviations in personal opinions and desires, which can trigger conflicts. Especially during the Covid-19-outbreak a bigger household size could increase stress at home, when the already limited supplies of food and income^28^ had to be shared among all family members.

Wanting a different amount of children can be a potential motive to begin a confrontation which ends in a rather violent way. Furthermore, it can be a reflection of generally differing personal preferences or ideas on family planning and childcare, which are again possible sources of conflicts.

This study highlights that victims feel discouraged from reporting physical abuse when their partners are present during the questions on “wife beating.” Underlying factors are cultural norms and societal expectations regarding gender roles as well as beliefs that a man has the right to discipline his partner or that women should submit to their husbands. Again, this indicates the need to discuss, question and shape beliefs which justify or normalize wife beating.

### Limitations

It is possible that our results underestimate the true number of IPV victims in Burkina Faso when respondents denied IPV for reasons of stigma or shame. The cross-sectional design does not allow any causal relationship, only an association between socio-demographic factors and the occurrence of IPV.

The different forms of IPV are based on specific questions created by the DHS program. Every other possible way of abuse which is not assessed by these questions could therefore be missed. The definition of wife rank varies according to the source. Here the time of marriage defines the rank among co-wives. However, according to different definitions the wife’s age, her socioeconomic status or her quality traits could as well be used to define the order. It is recommended to define questions and answers more clearly in future revisions of the DHS program.

No DHS dataset from Burkina Faso was collected between 2010 and 2021. Lacking a dataset which was collected in a comparable way to ours, it is challenging to assess whether the increase in IPV was a slow development since 2010 or a sudden increase related to the Covid-19-virus outbreak.

## Conclusion

Intimate partner violence against women is a phenomenon of multifactorial etiology that was highly prevalent in Burkina Faso in 2021. In order to address IPV effectively we suggest local authorities and human right organizations to focus on intimate partners that present with one or more of the associated socio-demographic characteristics. Whether the COVID-19-pandemic at that time was a direct cause or a coinciding factor remains unclear. Because of the novelty of COVID-19-related effects future research including longitudinal data would be highly appreciated.
